# Risks Perceived by Frail Male Patients, Family Caregivers and Clinicians in Hospital: Do they Change after Discharge? A Multiple Case Study

**DOI:** 10.5334/ijic.4166

**Published:** 2019-02-18

**Authors:** Véronique Provencher, Monia D’Amours, Chantal Viscogliosi, Manon Guay, Dominique Giroux, Véronique Dubé, Nathalie Delli-Colli, Hélène Corriveau, Mary Egan

**Affiliations:** 1 School of Rehabilitation, Faculty of Medicine and Health Sciences, Université de Sherbrooke and Research Centre on Aging, Québec, CA; 2 Research Centre on Aging, Québec, CA; 3 Department of Rehabilitation, Faculty of Medicine, Université Laval and Centre of Excellence on Aging, Québec, CA; 4 Research Centre, Centre hospitalier de l’Université de Montréal (CRCHUM), Québec, CA; 5 School of Social Work, Faculty of Arts, Humanities and Social Sciences, Université de Sherbrooke and Research Centre on Aging, Québec, CA; 6 School of Rehabilitation, Faculty of Health Sciences, University of Ottawa, Ontario, CA

**Keywords:** discharge planning, frail older adults, risk assessment, hospitalisation, integrated care, falls

## Abstract

**Introduction:**

Up to 40% of hospitalised seniors are frail and most want to return home after discharge. Inaccurate estimation of risks in the hospital may lead to inadequate support at home. This study aimed to document convergences and divergences between risks and support needs identified before hospital discharge and perceived at home post-discharge.

**Methods:**

This research used a multiple case study design. Three cases were recruited, each involving a hospitalised frail patient aged 70+, the main family caregiver and most of the clinicians who assessed the patient before and after hospital discharge. Thirty-two semi-structured interviews were conducted and their transcripts analysed using a qualitative thematic analysis approach.

**Results:**

Among risks raised by participants, falls were the only one with total inter-participant/inter-time/inter-case convergence. In all cases, all participants mentioned, before and after discharge, home adaptations and use of technical aids to mitigate this risk. However, clinicians recommended professional services while patients and family caregivers preferred to rely on family members and their own coping strategies.

**Conclusion:**

The divergences identified for most risks and support needs between users and clinicians, before and after discharge, provide new insights into a comprehensive and patient-centred risk assessment process to plan hospital discharge for frail elderly.

## Introduction

Frailty is a state of increased vulnerability, often associated with comorbidity and disability in the aging population [1]. Up to 40% of hospitalised older adults are frail [2] and most wish to return home after discharge [3]. However, during hospitalisation, functional decline [4,5] and deconditioning [6] often result in precarious discharge situations for frail patients [7], with increased risks of potentially preventable harm upon return home (e.g. falls, injuries, inadequate nutrition/medication intake) [8,9,10,11]. Recent data suggest these potential harms are largely preventable through optimal discharge planning [7,12,13].

Optimal discharge planning aims to ensure the continuity of quality care between hospital and community, and good coordination of services following discharge from hospital [14]. Discharge planning usually includes a risk assessment, in terms of both services and equipment, by an interdisciplinary team. Risk assessment prior to discharge is a complex process and clinicians in the hospital have difficulty accurately assessing what the “real” risks at home will be. The risk analysis process may be compromised by: 1) the patient’s unfamiliarity with the hospital context in which the assessment is done [15]; 2) the clinician’s lack of information about home hazards [16]; 3) fluctuations in the patient’s functional abilities due to medication, fatigue or pain [17]; and 4) the clinician’s difficulty predicting clinical progress after discharge, such as a sudden deterioration [5] or gradual improvement [18].

Underestimating risk can result in providing inadequate support for frail patients and their family caregivers after discharge. Unidentified risks and unmet support needs may lead to further functional decline [19], caregiver distress [20], unplanned hospitalisations [21], nursing home admissions [22], and even death [23]. Conversely, overestimating risk and recommending too much support may impede autonomy [24] and generate unnecessary costs [25]. Shorter hospital stays [26] and the variability of timely community health care follow-up [8,27] further underscore the need to identify and mitigate potential risks prior to hospital discharge.

Consequently, it is important to ensure that the risks and support needs identified prior to hospital discharge accurately reflect the risks faced and support needed after returning home. However, it is not known to what extent risk assessments, even when performed by clinicians at home, can help to estimate the risks and support needs of frail older adults and their family caregivers in their daily lives. It is thus of primary importance to include the points of view of patients and family caregivers concerning perceived risks throughout the 24-hour daily routine [19,28,29]. Patients and family caregivers could also provide unique information about factors that may increase or lessen the risks identified by clinicians, such as patients’ past habits [30] or family caregivers’ readiness to offer support [31,32]. While previous studies [28,33,34] considered perception of safety issues before and after discharge, to our knowledge none combined the perceptions of patients and family caregivers with those of most clinicians involved in risk assessments. It is vital to accurately document the extent to which risks and needs for support assessed prior to discharge match those assessed following discharge, based on the perceptions of patients, family caregivers and clinicians.

## Objective

In order to optimise planning for discharge to a safe home environment, this study aimed to inform risk assessment by exploring convergences and divergences between: 1) risks and support needs identified before hospital discharge and perceived at home post-discharge, and 2) health care users (patients and family caregivers) and clinicians involved in the patient evaluation (physicians, occupational therapists, physiotherapists, social workers, nurses and nutritionists).

## Methods

**Figure 1:**
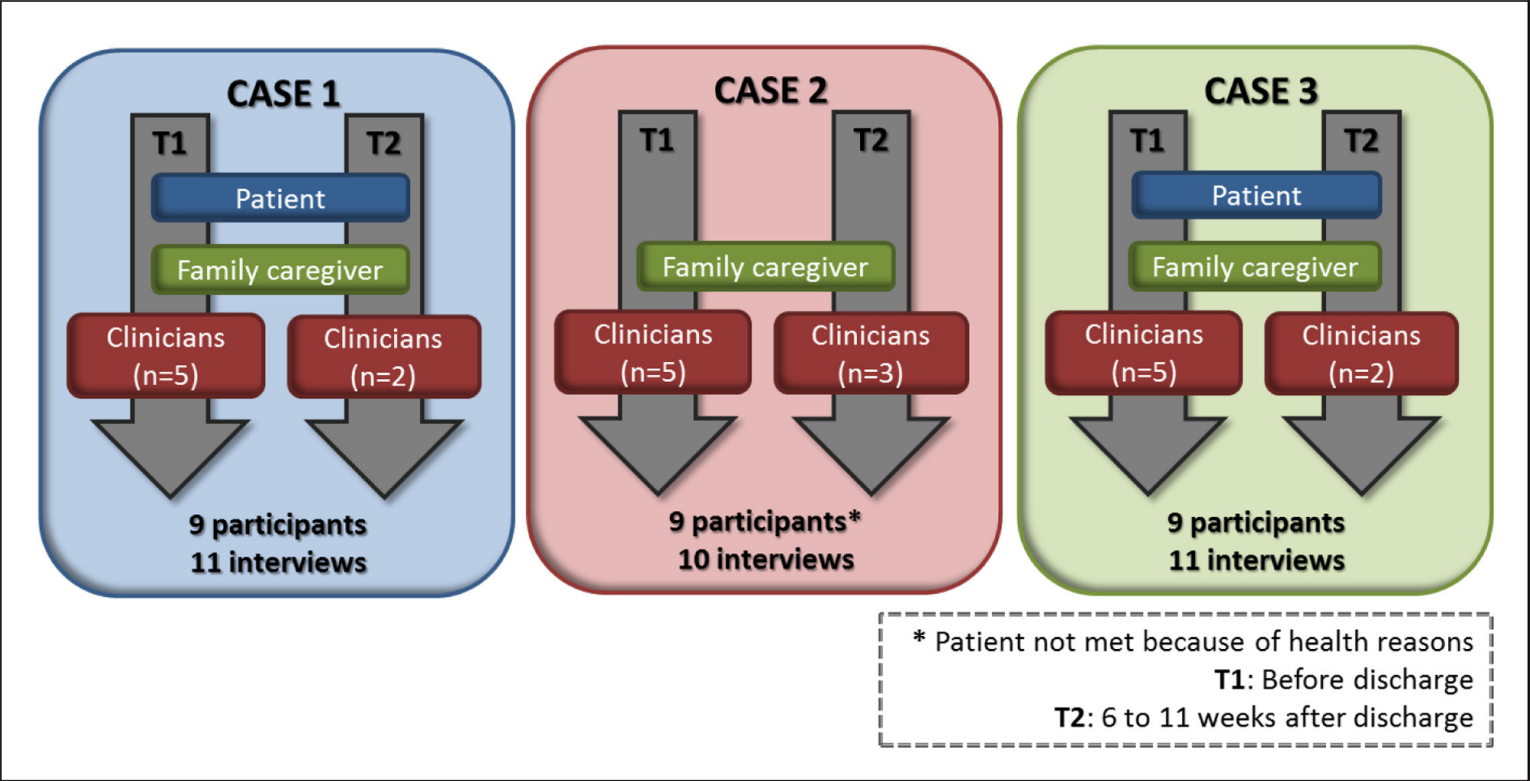
Methodological framework: multiple case study design.

This qualitative study used a multiple case study design [35]. This is an appropriate approach to shed light on clinical challenges in real contexts by expanding knowledge about patients’, families’ and clinicians’ perceptions of risks and support needs before and after hospital discharge (see Figure 1).

### Setting and participants

Three cases were recruited between October 2016 and February 2017 through an Intensive Functional Rehabilitation Unit (IFRU) in a semi-urban area (Sherbrooke, Quebec, Canada). In these settings, decisions surrounding hospital discharge are made by a multidisciplinary team. A research assistant scrutinised the medical files with the Unit’s head nurse to identify eligible patients. Each case had to involve: 1) a frail older patient or the main family caregiver; and 2) at least one clinician (occupational therapist, physician, physical therapist, social worker, nurse and/or nutritionist) who assessed the patient before and at least one clinician who assessed the patient after discharge (see Figure 1). To be eligible for the study, patients had to be aged 70 and over, speak French or English, have a family caregiver who could observe risks over a 24-hour period, and be evaluated by at least one hospital-based and at least one community-based clinician, respectively, before and after discharge. Patients were considered “frail” according to the Iso-SMAF score (profile 5, 6 or 9). The Iso-SMAF profile is a valid and reliable classification [36] based on the functional autonomy measurement system (SMAF) [37,38,39]. Briefly, patients needed help with activities of daily living but had to present intact cognition or only minor alterations in mental functions, with or without difficulties in mobility (see Appendix 1 for characteristics of the three profiles considered in the study). These criteria are akin to levels 5 and 6 of the Frailty Scale [40].

For ethical reasons, potential participants were approached in person by the Unit’s head nurse, who was not involved in the research project or patients’ direct care. Interested candidates had to give their verbal consent for a research assistant to contact them to verify eligibility criteria and agree on a time for the in-person interview. To recruit a case, the patient or family caregiver had to consent, as well as at least one hospital-based clinician and one community-based clinician. This research project was approved by the Ethics Committee of the CIUSSS de l’Estrie-CHUS.

### Data collection

Data were collected by a research assistant who had experience with qualitative approaches. Individual semi-structured interviews were conducted before discharge (T1) with the patient, family caregiver and hospital-based clinicians, and about six weeks after discharge (T2) with the patient, family caregiver and community-based clinicians. An interview grid was used to collect information on: a) perceived risks at home for the patient (e.g. “Which activities do you do during the day that worry you more than others? Why? Which ones are more stressful for your loved one/caregiver? Why? Can you describe one of your typical days? What do you do from the time you get up in the morning until you go to bed at night? What do you do during the night?”), and b) support (services, assistive devices, strategies) to offer to reduce those risks (e.g. “Are you already receiving some of the following services such as Meals on Wheels or help with bathing? If not, would you be willing to receive the service? If not, why not? What could be done to improve this situation?”). Interviews were audiotaped and transcribed in full. Sociodemographic data were collected through chart reviews. Patient records were also consulted to collect information on support provided at discharge.

### Data analysis

Interview transcripts were analysed by a senior research professional using qualitative content analysis, which is considered a comprehensive and suitable approach for case study research [41]. Following a thematic analysis process, transcripts were analysed line by line based on perception of risk and required assistance as units of analysis. Recurring, converging and diverging themes/patterns of risk and assistance were identified, along with illustrative examples. To identify relevant trends when documenting the risk assessment process, each case first underwent an internal analysis (intra-case) between: 1) pre- and post-discharge (inter-measuring time analysis); and 2) patient, family caregiver and clinicians (inter-participant analysis). A cross-case (inter-case) analysis was then conducted to target elements of convergence and divergence between the three cases. When performing the inter-case analysis, data pertaining to patients and family caregivers were merged into a single group to reflect users’ (patient-centred) perceptions compared to clinicians (also considered as a group). Validation of themes/patterns was performed by the main researcher through analysis of the interview transcripts. In the event of a disagreement, consensus was sought.

## Results

### Participants’ characteristics

Table 1 shows the participants’ characteristics for each of the three cases in the study. Each case involved nine participants (patient, family caregiver and clinicians). Patients were all men, the first being 71 years old while the other two were 84. Family caregivers were exclusively women, two spouses and one daughter, aged 70, 81 and 57, respectively. A total of 15 clinicians were met before discharge, and seven after discharge. They ranged from 27 to 59 years old, and 82% were women. Clinicians had a wide range of experience, with between 1½ and 35 years in the profession, and from three weeks to 30 years in the organisation. Clinicians involved before and after discharge were not the same.

**Table 1:** Participants’ characteristics in each case in the study.

Participants	Characteristics	CASES		
		1	2	3

Patient	Gender	Male	Male	Male
	Age	71	84	84
	Living situation	With wife, own home	With wife, own home	Alone, own home
	Living environment	Rural	Urban	Rural

Family caregivers	Gender	Female	Female	Female
	Age	70	81	57
	Link with patient	Spouse	Spouse	Daughter

Clinicians	T1-Number (Professions)	5 (Phy, OT, Physio, SW, Nut)	5 (Phy, OT, Physio, Nur, Nut)	5 (Phy, OT, Physio, SW, Nut)
	T2-Number (Professions)	2 (OT, SW)	3 (Pra, OT, Phy)	2 (OT, SW)
	Age range (years)	27–59	29–59	28–59
	Gender	2 Males, 5 Females	8 Females	2 Males, 5 Females
	Experience in profession	1½–24 years	4–35 years	2½–24 years
	Experience within organisation	9 months–24 years	6 months–30 years	3 weeks–24 years

Phy: Physician; OT: Occupational Therapist; Physio: Physiotherapist; SW: Social Worker; Nur: Nurse; Nut: Nutritionist.

A synopsis of the patients’ pathways before and after hospital discharge is presented in Table 2. All three patients lived in their own homes at the time of recruitment, two with spouses (who provided help with activities of daily living) and one alone (relying on friends for activities outside the home). All were hospitalised for surgery and had at least three comorbidities. The length of hospital stay was five to ten weeks. Once discharged, two patients showed a decline in their physical and mental health while the third greatly improved his walking ability and showed better mood. Only the case 1 patient was readmitted to hospital after the first discharge because of a severe rheumatoid arthritis crisis. Due to health problems, the case 2 patient could not meet with the research team.

**Table 2:** Synopsis of patients’ pathways before and after hospital discharge.

Pathway	CASE 1	CASE 2	CASE 3

BEFORE DISCHARGE			
Cause of hospitalisation	Hip surgery (vascular necrosis)	Hip surgery (post-fall fracture)	Lumbar surgery (severe spinal stenosis)
Comorbidities	Rheumatoid arthritis, diabetes, pulmonary embolism	Major depression, deconditioning, malnutrition	Bilateral neurapraxia, fall on shoulder, COPD
Past fall event	Yes	Yes	Yes
***T1 – STUDY***	**IFRU**	**IFRU**	**IFRU**

**DISCHARGE**			
Length of stay	10 weeks	5 weeks	10 weeks
**AFTER DISCHARGE**			

Physical health evolution*	Decrease	Decrease	Increase (walking ability)
Mental health evolution*	Decrease	Decrease	Increase
Health events	2 rheumatoid arthritis crises		One fall
Functional autonomy*	Decrease, dependent on spouse	Decrease, dependent on spouse	Depends on others for activities outside the home but very active
Social environment*	Mainly spouse but can count on some neighbours	Not interested in human contact	Family lives far away but he has many friends close by
**Readmission**	Yes (2×)	No	No
Time until readmission and length of stay	10 days later, for 2 weeks		
	13 days later, for >5 weeks		
***T2 – STUDY***	IFRU (11 weeks after discharge)**	**Home (7 weeks after discharge)**	**Home (6 weeks after discharge)**

* based on interviews; ** patient was readmitted to IFRU after discharge, but was interviewed at T2 concerning the time he was at home; COPD: Chronic Obstructive Pulmonary Disease; IFRU: Intensive Functional Rehabilitation Unit.

### Perceived risks

**Figure 2:**
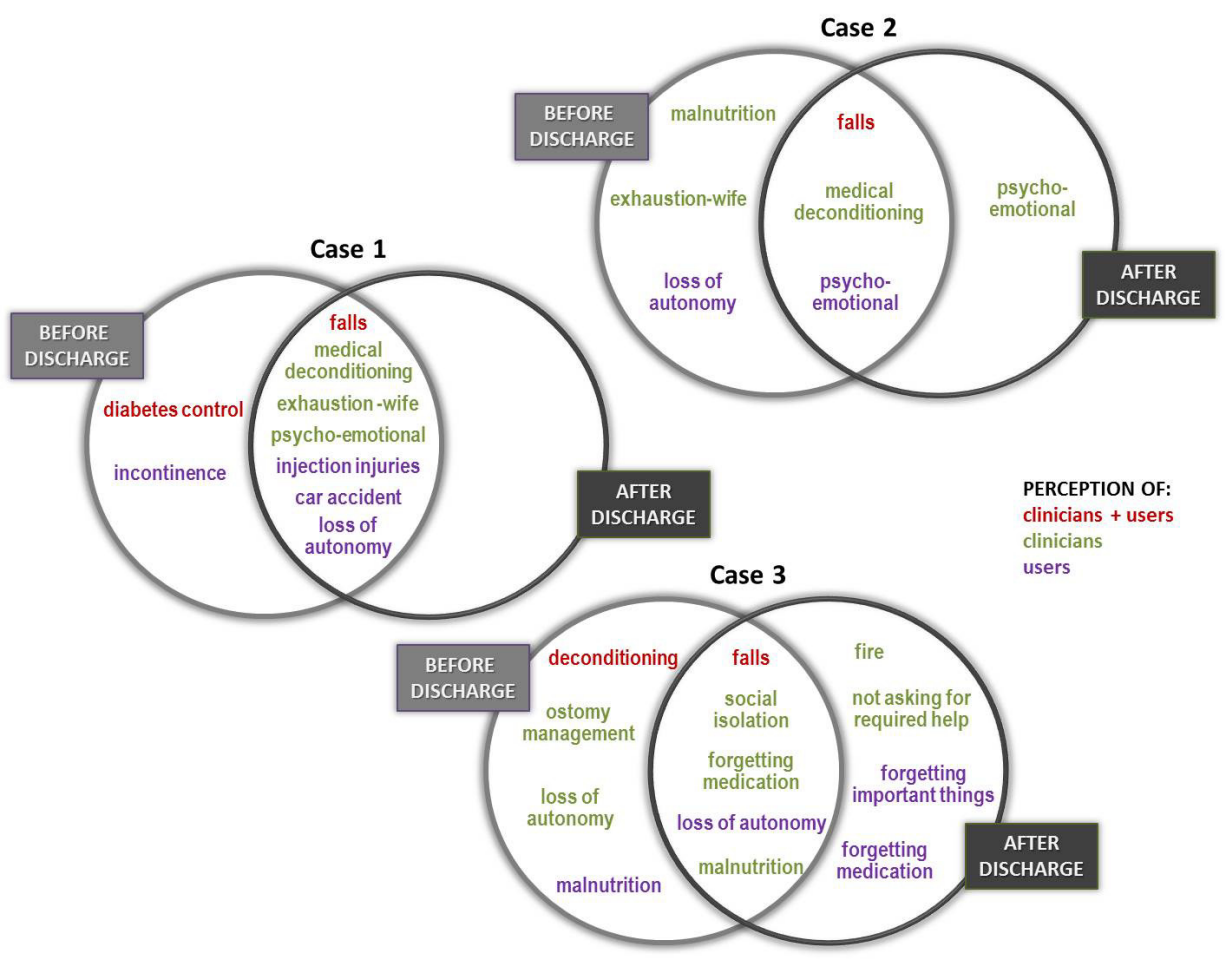
Overview of perceived risks – Inter-participant, inter-measuring time and inter-case comparison.

An overview of the risks perceived by patient, family caregiver and clinicians in each case, both before and after discharge, is presented in Figure 2.

First, falls were the only risk with total inter-participant, inter-time and inter-case convergence. Falls were clearly recognised as a risk before and after discharge by patients, family caregivers and clinicians in all three cases.

Loss of autonomy and medical deconditioning were two risks with partial convergence as they were mentioned before and after discharge in all cases but only by some of the participants. Loss of autonomy was mostly perceived by patients and caregivers, while medical deconditioning was often mentioned by clinicians. For example, a family caregiver said:

“You know, here [hospital], he just wheels up and it’s the right height for him. At home, things aren’t the right height for him. So I don’t know how it’s gonna be for him in the morning to get his things done.”

At the same time, a clinician talked about his concern regarding his patient’s medical deconditioning:

“There was a risk of deconditioning […] It was really his medical condition that I was apprehensive about […] about his physical health […] What happens is that his level of pain increases, the risk of inflammation, his physical condition evolves, his medical condition, and then there’s a breaking point and he calls an ambulance and goes to the hospital.”

Similarly, malnutrition, exhaustion of the wife and psycho-emotional risks partially converged at each level (inter-participant, inter-time and inter-case). Clinicians addressed these risks, which were hardly mentioned by patients or family caregivers, in two of the three cases. More specifically, malnutrition was raised by clinicians in case 2 (before discharge) and case 3 (before and after discharge), while exhaustion of the wife and psycho-emotional risks were mentioned before and after discharge by clinicians in both cases where the patient lived with a spouse (1 and 2). Only users in case 1 identified malnutrition before discharge, whereas users in case 2 targeted solely psycho-emotional risk before and after discharge.

Other risks mentioned by participants did not show any convergence, which does not mean that they are less important, just that they are specific to the case’s living situation and often perceived by a single group of participants. For example, car accidents and diabetes injection injuries were addressed before and after discharge but only by the patient/family caregiver in case 1. However, the spouse in case 2 perceived as stressful the risk of injuries when her husband was giving himself diabetes injections, a risk that was not identified by clinicians:

“… but he was coming to give it, with his fingers that are not really skilled. Sometimes he hurts himself with the syringe. I didn’t like it at all. It was stressing me a lot”.

**Figure 3:**
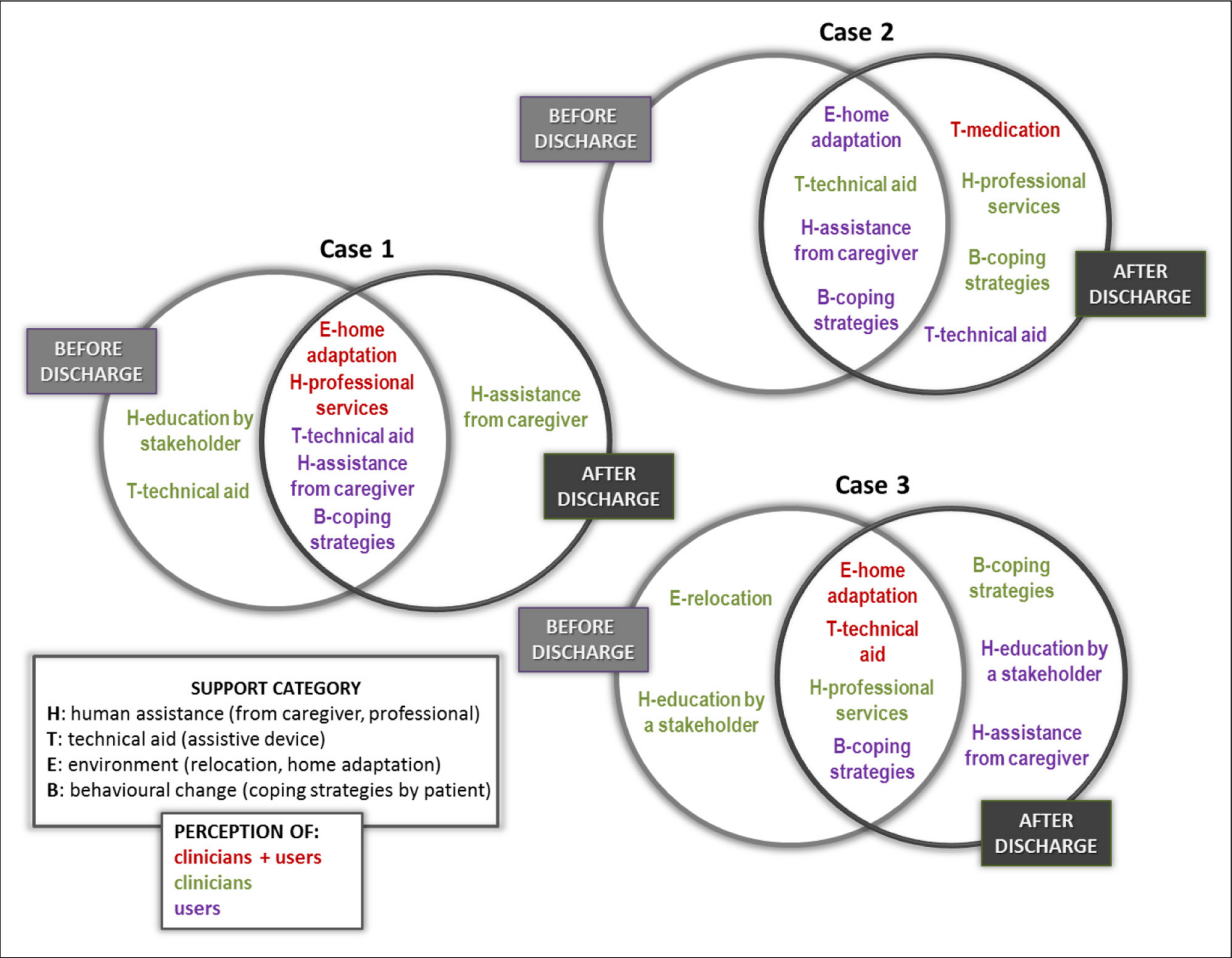
Perceived support for fall risk – Inter-participant, inter-measuring time and inter-case comparison.

Some other risks (fire, social isolation, not asking for required help, forgetting important things and ostomy management) were only related to case 3 and perceived mainly by clinicians (see Figure 3).

### Perceived support

Not surprisingly, falling is the risk that garnered the greatest number of different types of support, with eight types related to the four major categories (human assistance, technical aid, environment and behavioural change). As a lot of information is contained for falls, Figure 3 shows perceived support for this specific risk in a way that is easier to understand. One type of support to reduce the risk of falls – use of an assistive device (e.g. walker) – shows overall inter-case and inter-participant convergence. Thus, all participants in the three cases perceived the use of assistive devices as relevant. This applies before and after discharge to both groups of participants.

Home adaptation (e.g. remove stairs) is another important support to mitigate fall risk perceived by the two groups of participants in all cases both before and after discharge, except for clinicians in case 2. Otherwise, inter-case convergence was identified for professional services in one group (clinicians), and assistance from family caregiver and coping strategies used by patient in the other group (users). Professional services were mentioned before and after discharge by clinicians in cases 1 and 3, and after discharge only by clinicians in case 2. Support from family caregivers and using own coping strategies were named before and after discharge by users in cases 1 and 2, and after discharge only by users in case 3. For example, a patient mentioned that to minimise the risk of falls, he adapted the way he moved to ensure he had something to hold onto:

“I mean with the kitchen counter and all that, I just, you know, I’m not crazy, I won’t go walking in the middle of the living room. But I will walk in a way that, if something happens, I can hold onto a piece of furniture”.

For loss of autonomy, partial inter-case convergence was found for assistance from a family caregiver, and professional services and vehicle adaptation by users only, as clinicians in cases 1 and 2 did not mention this risk. Assistance from a family caregiver converged between users in cases 1 and 3 before and after discharge, while professional services were common to users in cases 2 and 3, but before discharge for the former and at both measuring times in case 3. Vehicle adaptation was addressed to reduce loss of autonomy by users in cases 2 and 3 after discharge only.

The groups of participants who referred to malnutrition (clinicians in cases 2 and 3, and users in case 3) mentioned the support by professional services to minimise it. This was before and after discharge for participants in case 3, and before discharge only for clinicians in case 2. Clinicians in these two cases also perceived education or training interventions as relevant but before discharge only. Professional services were also mentioned before and after discharge by clinicians in case 1 to reduce deconditioning, exhaustion of the wife and psycho-emotional risks, and by clinicians in case 2, before discharge for deconditioning and after discharge for psycho-emotional risk. To illustrate this result, a clinician in case 1 mentioned that setting up home services and alternative resources for the patient (e.g. transportation) would reduce caregiver’s burden:

“I think that setting up services would take a little weight off the shoulders of the spouse who has to compensate. It will help at this level. And to see different resources that could … for example, for transportation, instead of it always being the spouse who has to provide them, maybe we could … we will look for resources to … find other alternatives”.

## Discussion

This multiple case study identified convergences and divergences between risks and support needs targeted before hospital discharge and perceived after discharge by patients, family caregivers and clinicians. One important convergence pertained to the risk of falls, which was recognised as important by patients, family caregivers and clinicians, both before and after discharge. Malnutrition, on the other hand, was mostly addressed by clinicians only. Among other divergences found, loss of autonomy was mostly perceived by patients and family caregivers, while medical deconditioning was mainly reported by clinicians. Exhaustion of the wife was perceived before and after discharge solely by clinicians. Finally, clinicians recommended professional services, while patients and family caregivers preferred to rely on family members and their own coping strategies.

Total convergence regarding fall risk may be because all the patients had a fall event prior to hospitalisation and the perception of a risk may be strongly related to past events. MacLeod and Stadnyk [42] discussed the imminence of potentially harmful events in terms of whether dangerous events that had already happened increased the perceived risk. An imminent danger, such as falls for the patients in our study, would be more likely to be rated as “high risk” than more insidious threats that develop over a longer period of time, like malnutrition.

The risks of medical deconditioning and loss of autonomy are related since one (loss of autonomy) is a consequence of the other (medical deconditioning), yet there is an important nuance in the way each group of participants saw these risks. This may be because knowledge about patients’ ability to move when they need and want to throughout a 24-hour day (autonomy) is difficult for clinicians to capture, just as it is hard for patients to access information related to their precarious health status (deconditioning). According to previous studies [43,44,45], the perceptions of patients and family caregivers are not given enough consideration by clinicians when they evaluate risks and plan hospital discharges, whereas the patient input would be significant during these stages [46]. Close communication between clinicians and users before discharge is very important when making recommendations that target risks users view as important (loss of autonomy) as well as to improve patients’ awareness of risks regarding their health status (deconditioning, malnutrition).

Surprisingly, only clinicians mentioned exhaustion of the spouse before and after discharge. This suggests that patients may not fully recognise the burden they put on them. Furthermore, family caregivers do not seem to perceive the risk to their own health. One potential explanation is that spouses may see their role differently from children – as “part of the job” – rather than a burden [47]. Since exhaustion of family caregivers is known to be a major cause of rehospitalisation following discharge [44,48], it is essential to take upstream action to reduce risks perceived as stressful by family caregivers [31,32,49]. Support that aligns with their specific needs (such as patient training to avoid injection injuries) could do more to ease their stress than generic services [50].

The fact that almost two thirds of the risks (10/16) mentioned by participants did not show any inter-case or inter-participant convergence highlights the importance of considering the particular characteristics of each case. For example, car accidents were mentioned by patient and family caregiver in one case before and after discharge but never by clinicians, while fires were addressed only by clinicians in another case after discharge. This gives cause for concern as underestimating or overestimating these risks, although uncommon, can lead to serious consequences (loss of driver’s license, relocation, injuries, or even death) for the patient, family caregivers or others [51], depending on the context.

Overall, our results point to the need to review the way risks are assessed and addressed, especially those that are hard to capture but are important to consider when trying to improve patients’ and others’ quality of life and safety. An enhanced patient-centred approach [52] using an interview guide [53], which may include open-ended questions such as “What are you worried about?”, or systematically involving patients and families in interdisciplinary meetings before discharge [54,55], may reveal risks that can only be identified by patients or family caregivers (car accidents, injection injuries). This process may also help to understand whether some unidentified risks (malnutrition, exhaustion of the wife) are considered “acceptable” to patients, and work with them to find suitable support to lessen their potential impacts [56].

Our findings also raise the issue of the acceptability of the support recommended, which varies between groups of participants. Human assistance provided by professional services is more likely to be recommended by clinicians while patients prefer to ask for assistance from family caregivers or make changes in their own behaviour. Showing a preference for seeking help from family caregivers is in line with previous studies conducted with older adults (see systematic review by Werner et al., 2014) [57]. Relying on external help may be considered an indication of dependency by patients or family caregivers, as well as involving the discomfort of being helped by a stranger [58]. This result underscores the importance of clinicians ensuring the acceptability for patient and family caregiver of support recommended at discharge by building a partnership to look for a compromise that suits their needs and preferences. Making recommendations that are appropriate but unacceptable to patients may be useless and explain how little positive impact they have on mitigating risks at home [59]. To reach a compromise, clinicians should initiate dialogue with patient and family caregiver to seek acceptable solutions. Temporary hospital discharges, which are often embedded in the discharge planning process, may also be used to document if new difficulties arise while at home for a few days, and if patients change their minds about perceived support needs [60].

### Strengths and limitations of the study

First, due to health problems, the case 2 patient could not meet with the research team. This led to missing information about the patient’s perception of risks and support needs in this case. However, the user’s perceptions were obtained from a caregiver living with him, who was very familiar with the risks faced by this patient on a daily basis.

Second, there was only a small number of cases, all admitted to the same Intensive Functional Rehabilitation Unit, which may limit the external validity of the study. However, gathering information for each case from nine participants with different perspectives (patient, family caregiver and clinicians from different disciplines and with a wide variety of experience) provided a wealth of information regarding perceived risks before and after hospital discharge. Case studies also have the advantage of examining data from real life situations at the micro-level, which provides better insights into the behaviours concerned [61]. Our results may not be applicable to frail female patients. Since sex was not a criterion to exclude a participant, and considering that recruiting frail elderly is particularly challenging, we included the three men in the study.

Finally, differences in “what constitutes a risk” may explain some of the discrepancies between participants [42]. This was partially overcome by asking users to report a typical day during the interviews [62] instead of questioning them directly about risks. However, questions do not provide information regarding whether a risk has been identified or is considered important by participants. It is also difficult to know to what extent variability over time is attributable to changes in perceptions (risk prioritisation) or in the patient’s condition. A risk perceived before discharge may still be present after discharge but changes in the acceptability of this risk may have evolved and led the user to perceive it as less important. We also based our analyses on the assumption that all identified risks were an issue, but we did not know (since patients were not followed later) whether any would lead to rehospitalisation, injury, death, etc.

## Conclusion

This study revealed many differences in how risks are perceived by patients, caregivers and clinicians, before and after hospital discharge, except for fall risks. These results will help clinicians determine the best pre-discharge decisions to meet support needs at home for patients and their families by providing new insights into a comprehensive and patient-centred risk assessment process. Accurate and timely identification of serious risks at home, taking into account patient and family risk tolerance, is crucial to reducing risks both effectively and acceptably. A good fit between support provided and actual needs is expected to impact positively on rates of hospital readmission, relocation to nursing homes, and caregiver quality of life as well as patient safety and autonomy. Further studies are needed to understand what could help patients accept needed services in order to ensure safety after discharge and to determine what factors are related to the acceptability of these services.

## References

[1] Fried, LP, Ferrucci, L, Darer, J, Williamson, JD and Anderson, G. Untangling the concepts of disability, frailty, and comorbidity: Implications for improved targeting and care. The Journals of Gerontology Series A, Biological Sciences and Medical Sciences, 2004; 59(3): 255–63. DOI: 10.1093/gerona/59.3.M255 15031310

[2] Joosten, E, Demuynck, M, Detroyer, E and Milisen, K. Prevalence of frailty and its ability to predict in hospital delirium, falls, and 6-month mortality in hospitalized older patients. BMC Geriatrics, 2014; 14(1). DOI: 10.1186/1471-2318-14-1 PMC3905102 24393272

[3] Wiles, JL, Leibing, A, Guberman, N, Reeve, J and Allen, RE. The meaning of “aging in place” to older people. The Gerontologist, 2011; 52(3): 357–66. DOI: 10.1093/geront/gnr098 21983126

[4] Covinsky, KE, Palmer, RM, Fortinsky, RH, Counsell, SR, Stewart, AL, Kresevic, D, et al. Loss of independence in activities of daily living in older adults hospitalized with medical illnesses: Increased vulnerability with age. Journal of the American Geriatrics Society, 2003; 51(4): 451–8. DOI: 10.1046/j.1532-5415.2003.51152.x 12657063

[5] Wu, HY, Sahadevan, S and Ding, YY. Factors associated with functional decline of hospitalised older persons following discharge from an acute geriatric unit. Annals of Academic Medicine Singapore, 2006; 35(1): 17–23. 16470269

[6] Timmer, AJ, Unsworth, CA and Taylor, NF. Rehabilitation interventions with deconditioned older adults following an acute hospital admission: A systematic review. Clinical Rehabilitation, 2014; 28(11): 1078–86. DOI: 10.1177/0269215514530998 24844238

[7] Bowles, KH, Hanlon, A, Holland, D, Potashnik, SL and Topaz, M. Impact of discharge planning decision support on time to readmission among older adult medical patients. Professional Case Management, 2014; 19: 29–38. DOI: 10.1097/01.PCAMA.0000438971.79801.7a 24300427 PMC4072205

[8] Greenwald, JL, Denham, CR and Jack, BW. The hospital discharge: A review of a high risk care transition with highlights of a reengineered discharge process. Journal of Patient Safety, 2007; 3(2): 97–106. DOI: 10.1097/01.jps.0000236916.94696.12

[9] Douglas, A, Letts, L, Eva, K and Richardson, J. Measurement of harm outcomes in older adults after hospital discharge: Reliability and validity. Journal of Aging Research, 2012; 7 DOI: 10.1155/2012/150473 PMC3357527 22649728

[10] Meyer, OL, Sisco, SM, Harvey, D, Zahodne, LB, Glymour, MM, Manly, JJ, et al. Neighborhood predictors of cognitive training outcomes and trajectories in ACTIVE. Research on Aging, 2017; 39(3): 443–67. DOI: 10.1177/0164027515618242 26667987 PMC4905811

[11] Yeh, C. Fostering a new (more self-empowering) world view on aging. Generations, 2015; 39(1): 10–4.

[12] Hunter, T, Nelson, JR and Birmingham, J. Preventing readmissions through comprehensive discharge planning. Professional Case Management, 2013; 18(2): 56–63. DOI: 10.1097/NCM.0b013e31827de1ce 23241896

[13] Sheridan, E, Thompson, C, Pinheiro, T, Robinson, N, Davies, K and Whitmore, N. Optimizing transitions of care – hospital to community. Healthcare Quality, 2017; 20(1): 45–9. DOI: 10.12927/hcq.2017.25135 28550700

[14] Shepperd, S, Parkes, J, McClaran, J and Phillips, C. Discharge planning from hospital to home (Review). Cochrane Database of Systematic Reviews, 2004; 1 DOI: 10.1002/14651858.CD000313.pub2 14973952

[15] Provencher, V, Demers, L, Gagnon, L and Gelinas, I. Impact of familiar and unfamiliar settings on cooking task assessments in frail older adults with poor and preserved executive functions. International Psychogeriatrics, 2012; 24(5): 775–83. DOI: 10.1017/S104161021100216X 22153134

[16] Ramsdell, J, Jackson, J, Guy, H and Renvall, M. Comparison of clinic-based home assessment to a home visit in demented elderly patients. Alzheimer Disease & Associated Disorders, 2004; 18(3): 145–53. DOI: 10.1097/01.wad.0000137863.90007.ec 15494620

[17] Campbell, AJ and Buchner, DM. Unstable disability and the fluctuations of frailty. Age and Ageing, 1997; 26(4): 315–18. DOI: 10.1093/ageing/26.4.315 9271296

[18] Inouye, SK, Zhang, Y, Han, L, Leo-Summers, L, Jones, R and Marcantonio, E. Recoverable cognitive dysfunction at hospital admission in older persons during acute illness. Journal of General Internal Medicine, 2006; 21(12): 1276–81. DOI: 10.1111/j.1525-1497.2006.00613.x 16965558 PMC1924736

[19] Andreasen, J, Lund, H, Aadahl, M and Sorensen, EE. The experience of daily life of acutely admitted frail elderly patients one week after discharge from the hospital. International Journal of Qualitative Studies on Health and Well-being, 2015; 10: 1 DOI: 10.3402/Fqhw.v10.27370 PMC4452652 26037333

[20] Bauer, M, Fitzgerald, L and Koch, S. Hospital discharge as experienced by family carers of people with dementia: A case for quality improvement. Journal for Healthcare Quality, 2011; 33(6): 9–16. DOI: 10.1111/j.1945-1474.2011.00122.x 22103700

[21] Depalma, G, Xu, H, Covinsky, KE, Craig, BA, Stallard, E, Thomas, J, et al. Hospital readmission among older adults who return home with unmet need for ADL disability. Gerontologist, 2013; 53(3): 454–61. DOI: 10.1093/geront/gns103 22859438 PMC3635854

[22] Boockvar, KS, Litke, A, Penrod, JD, Halm, EA, Morrison, RS, Silberzweig, SB, et al. Patient relocation in the 6 months after hip fracture: Risk factors for fragmented care. Journal of the American Geriatrics Society, 2004; 52(11): 1826–31. DOI: 10.1111/j.1532-5415.2004.52512.x 15507058 PMC1447596

[23] He, S, Craig, BA, Xu, H, Covinsky, KE, Stallard, E, Thomas, J, et al. Unmet need for ADL assistance is associated with mortality among older adults with mild disability. The Journals of Gerontology Series A, Biological Sciences and Medical Sciences, 2015; 70(9): 1128–32. DOI: 10.1093/gerona/glv028 PMC4841172 25834196

[24] Proot, IM, Crebolder, HF, Abu-Saad, HH, Macor, TH and Ter Meulen, RH. Stroke patients’ needs and experiences regarding autonomy at discharge from nursing home. Patient Education and Counseling, 2000; 41(3): 275–83. DOI: 10.1016/S0738-3991(99)00113-5 11042430

[25] Nygard, N, Grahn, U and Rudenhammer, A. Reflecting on practice: Are home visits prior to discharge worthwhile? Scandinavian Journal of Caring Sciences, 2000; 18: 183–203. 10.1111/j.1471-6712.2004.00270.x 15147483

[26] Tardif, I and Fleury, FC. Activités liées au congé dans la trajectoire de services pour les aînés en soins postaigus – Recension des écrits. [Activities related to discharge in the service trajectory for seniors in post-acute care – A literature review] Longueuil: Agence de santé et des services sociaux de la Montérégie; 2013. [in French].

[27] Raymond, MH, Demers, L and Feldman, DE. Waiting list management practices for home-care occupational therapy in the province of Quebec, Canada. Health and Social Care in the Community, 2016; 24(2): 154–64. DOI: 10.1111/hsc.12195 25684435

[28] Cain, CH, Neuwirth, E, Bellows, J, Zuber, C and Green, J. Patient experiences of transitioning from hospital to home: An ethnographic quality improvement project. Journal of Hospital Medicine, 2012; 7(5): 382–87. DOI: 10.1002/jhm.1918 22378714

[29] Kangovi, S, Grand, D, Meehan, P, Mitra, N, Shannon, R and Long, JA. Perceptions of readmitted patients on the transition from hospital to home. Journal of Hospital Medicine, 2012; 7(9): 709–12. DOI: 10.1002/jhm.1966 23212980

[30] Giroux, D, Tétreault, S and Langlois, L. Présentation d’un modèle décisionnel concernant l’aptitude d’une personne âgée atteinte de déficits cognitifs à gérer sa personne et ses biens. [Presentation of a decision-making model concerning the ability of an elderly person with cognitive deficits to manage his person and his property.] La Revue francophone de gériatrie et de gérontologie, 2012; XIX(186): 224–36 Tome. [in French].

[31] Giosa, JL, Stolee, P, Dupuis, SL, Mock, SE and Santi, SM. An examination of family caregiver experiences during care transitions of older adults. Canadian Journal on Aging, 2014; 33(2): 137–53. DOI: 10.1017/S0714980814000026 24754978

[32] Mockford, C. A review of family carers’ experiences of hospital discharge for people with dementia, and the rationale for involving service users in health research. Journal of Healthcare Leadership, 2015; 7: 21–8. DOI: 10.2147/JHL.S70020 29355178 PMC5740992

[33] Naylor, M, Stephens, C, Bowles, KH and Bixby, MB. Cognitively impaired older adults: From hospital to home. American Journal of Nursing, 2005; 105(2): 52–61. DOI: 10.1097/00000446-200502000-00028 15674058

[34] Shyu, YI. The needs of family caregivers of frail elders during the transition from hospital to home: A Taiwanese sample. Journal of Advanced Nursing, 2000; 32(3): 619–25. DOI: 10.1046/j.1365-2648.2000.01519.x 11012804

[35] Yin, RK. Case study research design and methods (5th ed.) Thousand Oaks, CA: Sage; 2014.

[36] Dubuc, N, Hébert, R, Desrosiers, J, Buteau, M and Trottier, L. Disability-based classification system for older people in integrated long-term care services: The Iso-SMAF profiles. Archives of Gerontology and Geriatrics, 2006; 42: 191–206. DOI: 10.1016/j.archger.2005.07.001 16125809

[37] Hébert, R, Carrier, R and Bilodeau, A. The functional autonomy measurement system (SMAF): Description and validation of an instrument for the measurement of handicaps. Age and Ageing, 1988; 17(5): 293–302. DOI: 10.1093/ageing/17.5.293 2976575

[38] Hébert, R, Guilbeault, J, Desrosiers, J and Dubuc, N. The functional autonomy measurement system (SMAF): A clinical-based instrument for measuring disabilities and handicaps in older people. Geriatrics Today: Journal of Canadian Geriatrics Society, 2001; 4: 141–47.

[39] Desrosiers, J, Bravo, G, Hébert, R and Dubuc, N. Reliability of the revised functional autonomy measurement system (SMAF) for epidemiological research. Age and Ageing, 1995; 24: 402–06. DOI: 10.1093/ageing/24.5.402 8669343

[40] Rockwood, K, Song, X, MacKnight, C, Bergman, H, Hogan, DB, McDowell, I, et al. A global clinical measure of fitness and frailty in elderly people. Canadian Medical Association Journal, 2005; 173(5): 489–95. DOI: 10.1503/cmaj.050051 16129869 PMC1188185

[41] Kohlbacher, F. The use of qualitative content analysis in case study research. Forum: Qualitative Social Research, 2006; 7(1): Art. 21.

[42] MacLeod, H and Stadnyk, R. Risk: ‘I know it when I see it’: How health and social practitioners defined and evaluated living at risk among community-dwelling older adults. Health, Risk & Society, 2015; 17: 46–63. DOI: 10.1080/13698575.2014.999749

[43] Allen, J, Hutchinson, AM, Brown, R and Livingston, PM. Discharge planning of stroke patients: The relatives’ perceptions of participation. Journal of Clinical Nursing, 2009; 18(6): 857–65. DOI: 10.1111/j.1365-2702.2008.02600.x 19239664

[44] Bauer, M, Fitzgerald, L, Haesler, E and Manfrin, M. Hospital discharge planning for frail older people and their family. Are we delivering best practice? A review of the evidence. Journal of Clinical Nursing, 2009; 18(18): 2539–46. DOI: 10.1111/j.1365-2702.2008.02685.x 19374695

[45] Fitzgerald, L, Bauer, M, Koch, S and King, S. Hospital discharge: Recommendations for performance improvement for family carers of people with dementia. Australian Health Review, 2011; 35(3): 364–70. DOI: 10.1071/AH09811 21871200

[46] Ohta, B, Mola, A, Rosenfeld, P and Ford, S. Early discharge planning and improved care transitions: Pre-admission assessment for readmission risk in an elective orthopedic and cardiovascular surgical population. International Journal of Integrated Care, 2016; 16(2): 10 DOI: 10.5334/Fijic.2260 PMC5015549 27616965

[47] Chappell, NL, Dujela, C and Smith, A. Spouse and adult child differences in caregiving burden. Canadian Journal on Aging, 2014; 33(4): 462–72. DOI: 10.1017/S0714980814000336 25247325

[48] Bonin-Guillaume, S, Durand, AC, Yahi, F, Curiel-Berruyer, M, Lacroix, O, Cretel, E, et al. Predictive factors for early unplanned rehospitalization of older adults after an ED visit: Role of the caregiver burden. Aging Clinical and Experimental Research, 2015; 27(6): 883–91. DOI: 10.1007/s40520-015-0347-y 25835219

[49] Toye, C, Parsons, R, Slatyer, S, Aoun, SM, Moorin, R, Osseiran-Moisson, R, et al. Outcomes for family carers of a nurse-delivered hospital discharge intervention for older people (the Further Enabling Care at Home Program): Single blind randomised controlled trial. International Journal of Nursing Studies, 2016; 64: 32–41. DOI: 10.1016/j.ijnurstu.2016.09.012 27684320

[50] Pinkart, M and Sorensen, S. Helping caregiver s of persons with dementia: Which interventions work and how large are their effects? International Psychogeriatrics, 2006; 18(4): 577–95. DOI: 10.1017/S1041610206003462 16686964

[51] Culo, S. Risk assessment and intervention for vulnerable older adults. BC Medical Journal, 2011; 53(8): 421–25.

[52] Egan, MY, Laliberte Rudman, D, Ceci, C, Kessler, D, McGrath, C, Gardner, P, et al. Seniors, risk and rehabilitation: Broadening our thinking. Disability and Rehabilitation, 2017; 39(13): 1348–55. DOI: 10.1080/09638288.2016.1192227 27291255

[53] Wallace, A, Papke, T, Davisson, E, Spooner, K and Gassman, L. Provider opinions and experiences regarding development of a social support assessment to inform hospital discharge: The going home toolkit. Professional Case Management, 2017; 22(5): 214–27. DOI: 10.1097/NCM.0000000000000234 28777234

[54] Almborg, AH, Ulander, K, Thulin, A, Berg, S and Baker, GR. Enhancing the continuum of care. Report of the Avoidable Hospitalization Advisory Panel. Submitted to the Ministry of Health and Long-Term Care; 2011.

[55] Hesselink, G, Flink, M, Olsson, M, Barach, P, Dudzik-Urbaniak, E, Orrego, C, et al. Are patients discharged with care? A qualitative study of perceptions and experiences of patients, family members and care providers. BMJ Quality & Safety, 2012; 21(Suppl 1): i39–49. DOI: 10.1136/bmjqs-2012-001165 23118410

[56] Durocher, E and Gibson, BE. Navigating ethical discharge planning: A case study in older adult rehabilitation. Australian Occupational Therapy Journal, 2010; 57: 2–7. DOI: 10.1111/j.1440-1630.2009.00826.x 20854559

[57] Werner, P, Goldstein, D, Karpas, DS, Chan, L and Lai, C. Help-seeking for dementia: A systematic review of the literature. Alzheimer Disease & Associated Disorders, 2014; 28(4): 299–310. DOI: 10.1097/WAD.0000000000000065 25321607

[58] Levine, C and Lee, T. “I Can Take Care of Myself!” Patients’ Refusals of Home Health Care Services. A report from a roundtable sponsored by United Hospital Fund and the Alliance for Home Health Quality and Innovation; 2017.

[59] Topaz, M, Kang, Y, Holland, D, Ohta, B, Rickar, K and Bowles, KH. Higher 30-day and 60-day readmission among patients who refuse post-acute care services. American Journal of Managed Care, 2015; 21(5): 424–33. 26168063 PMC8607532

[60] Mukherjee, D. Discharge decisions and the dignity of risk. Hastings Center Report, 2015; 45(3): 7–8. DOI: 10.1002/hast.441 25944199

[61] Zainal, Z. Case study as a research method. Jurnal Kemanusiaan, 2007; 9: 6.

[62] Lang, A, Macdonald, MT, Storch, J, Stevenson, L, Mitchell, L, Barber, T, et al. Researching triads in home care: Perceptions of safety from home care clients, their caregivers, and providers. Home Health Care Management Practice, 2014; 26: 59–71. DOI: 10.1177/1084822313501077

